# Supplementary material for: Electron microscopy analysis of ATP-independent nucleosome unfolding by FACT
Source: Commun Biol. 2022 Jan 10;5:2. doi: 10.1038/s42003-021-02948-8 (PMC8748794; doi:10.1038/s42003-021-02948-8)
Supplement: Supplementary file 1 — Supplemental Material [file 42003_2021_2948_MOESM1_ESM.pdf]

Electron Microscopy Analysis of

ATP-Independent Nucleosome Unfolding by FACT

Sivkina, Anastasia L., Karlova, Maria G., Valieva, Maria E., McCullough, Laura L., Formosa, Timothy, Shaytan, Alexey K., Feofanov, Alexey V., Kirpichnikov, Mikhail P., Sokolova, Olga S., Studitsky, Vasily M.

Supplementary Information

Supplementary Table 1. Quantitation of the open and closed FACT complexes with Nhp6 and nucleosomes.

Supplementary Table 2. Comparison of FACT (S/P) and FACT:Nhp6 complexes using the Fisher square test.

Supplementary Figure 1. Native PAGE analysis of S/P+Nhp6 complexes.

Supplementary Figure 2. Native gel analysis of WT and mutant FACT complexes.

Supplementary Figure 3. Subunits analysis of FACT complexes by SDS PAGE.

Supplementary Figure 4. 2D class-averages of Spt16/Pob3 (top) and Spt16/Pob3:Nhp6 complexes (bottom).

Supplementary Figure 5. A model of FACT and Nhp6:FACT complexes in closed and open states.

Supplementary Figure 6. Analysis of FACT-nucleosome complex by FRET-in-gel.

Supplementary Figure 7. 2D Class-averages of Spt16/Pob3:Nhp6:nucleosome complexes.

Supplementary Figure 8. 2D Class-averages of samples containing Nhp6 and nucleosome.

Supplementary Figure 9. Hypothetical model of a complex of tetrasome with M-domain of Spt16 subunit of yFACT.

Supplementary Figure 10. The proposed sequential pathway of nucleosome unfolding by FACT:Nhp6.

Supplementary Table 1. Quantitation of the open and closed FACT complexes with Nhp6 and nucleosomes. Fractions of open and closed complexes were calculated as the average of three experiments; the mean values -/+ SD are shown.

**Sample**	**N particles**	**% ≤90° (closed)**	**% >90° (open)**
**Spt16/Pob3**	10304	64,0±3,3	36,0±3,3
**Spt16/Pob3 + Nhp6**	28425	48,7±4,8	51,3±4,8
**N + Spt16/Pob3 + Nhp6**	2139	45,0±8,6	55,0±8,6
**N + Nhp6**	3251	100,0	NA

Supplementary Table 2. Comparison of FACT (S/P) and FACT:Nhp6 complexes using the Fisher square test. The Fisher exact test statistic p value is < 0.00001.

Class\Sample	S/P	S/P+Nhp6	***Marginal Row Totals***
<90°	6595	13843	20438
>90°	3709	14582	18291
Marginal Column Totals	10304	28425	38729 (Grand Total)

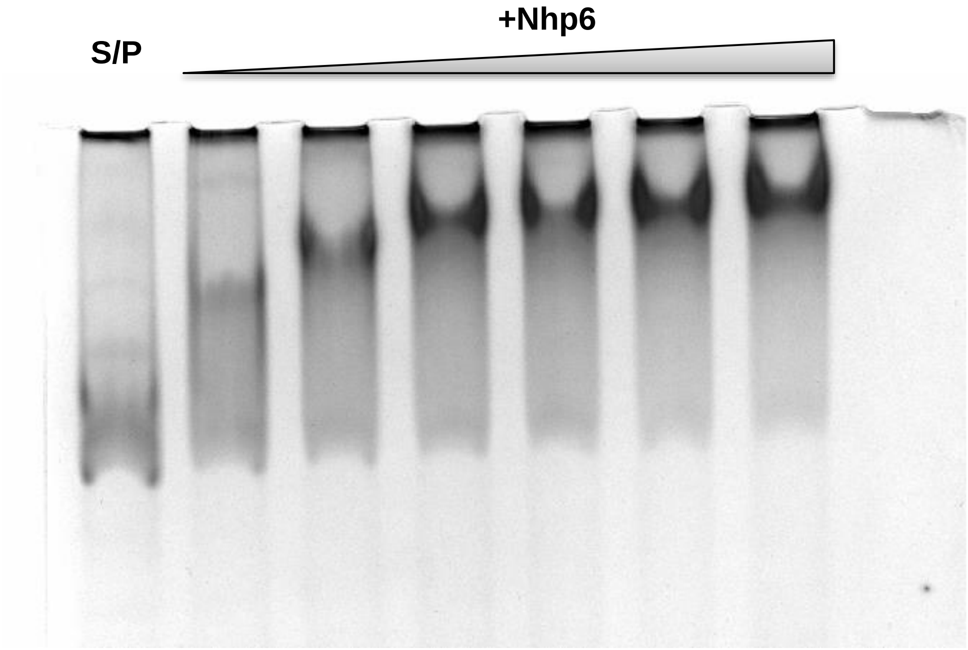

Supplementary Figure 1. Native PAGE analysis of S/P+Nhp6 complexes. Spt16/Pob3 (S/P, 0.13 µM) was incubated with Nhp6 (0, 0.26 µM, 0.52 µM, 0.78 µM, 1.04 µM,1.3 µM, or 2.6 µM) and analyzed by native PAGE followed by silver staining.

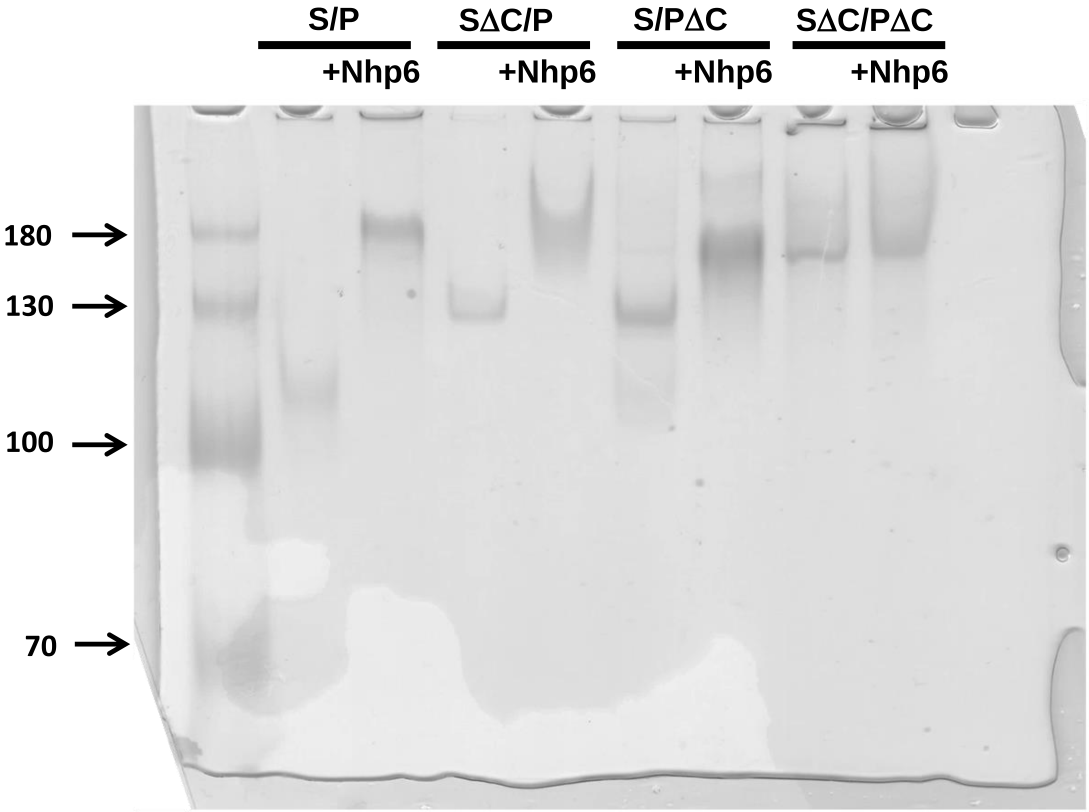

Supplementary Figure 2. Native gel analysis of WT and mutant FACT complexes. Native PAGE analysis of the migration of FACT mutants lacking the C-terminal regions of Spt16 (SΔC), Pob3 (PΔC), or both, with or without Nhp6, stained with Coomassie blue.

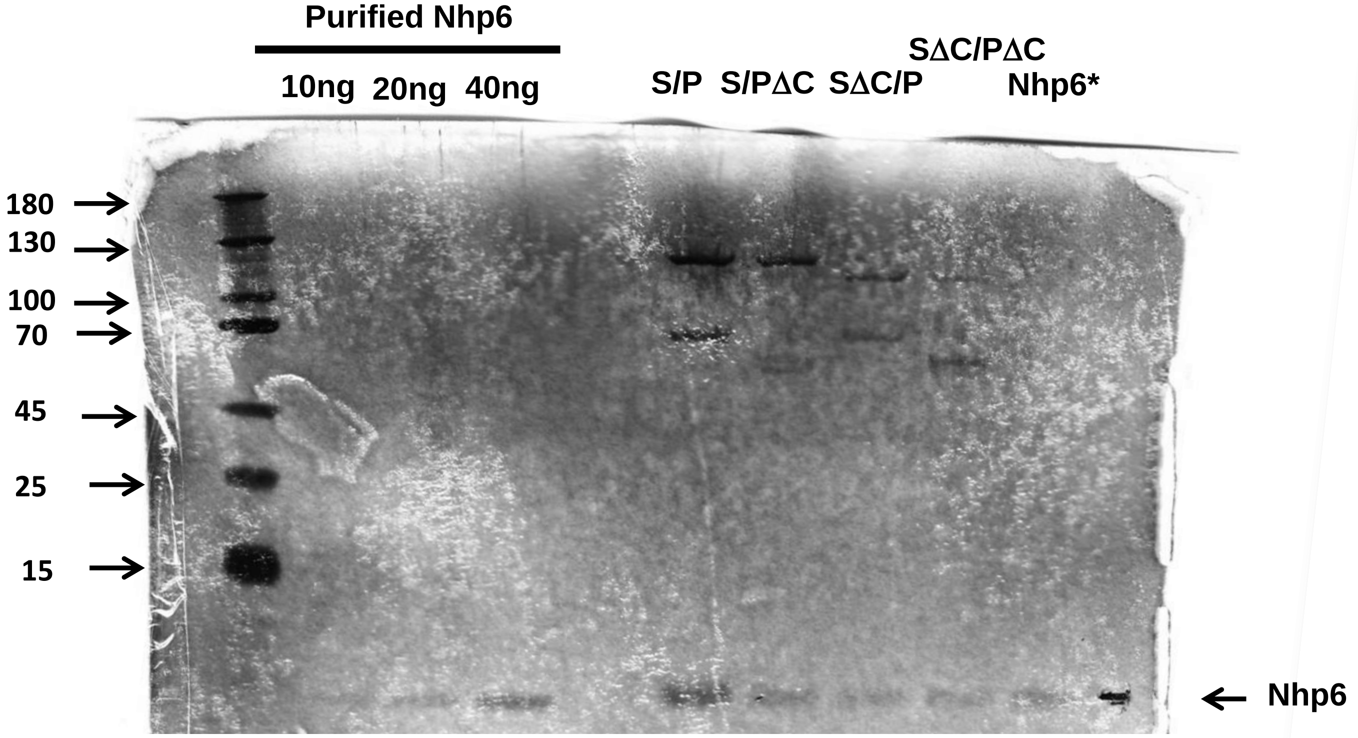

Supplementary Figure 3. Subunits analysis of FACT complexes by SDS PAGE. Bands (as in **1c**) containing apparent FACT:Nhp6 complexes were excised, subjected to denaturing SDS-PAGE and silver stained. The region of the gel containing Nhp6 protein is shown. Nhp6* shows Nhp6 level in the empty area of the gel from the lane containing Nhp6 only, indicating the background level of Nhp6 detection.

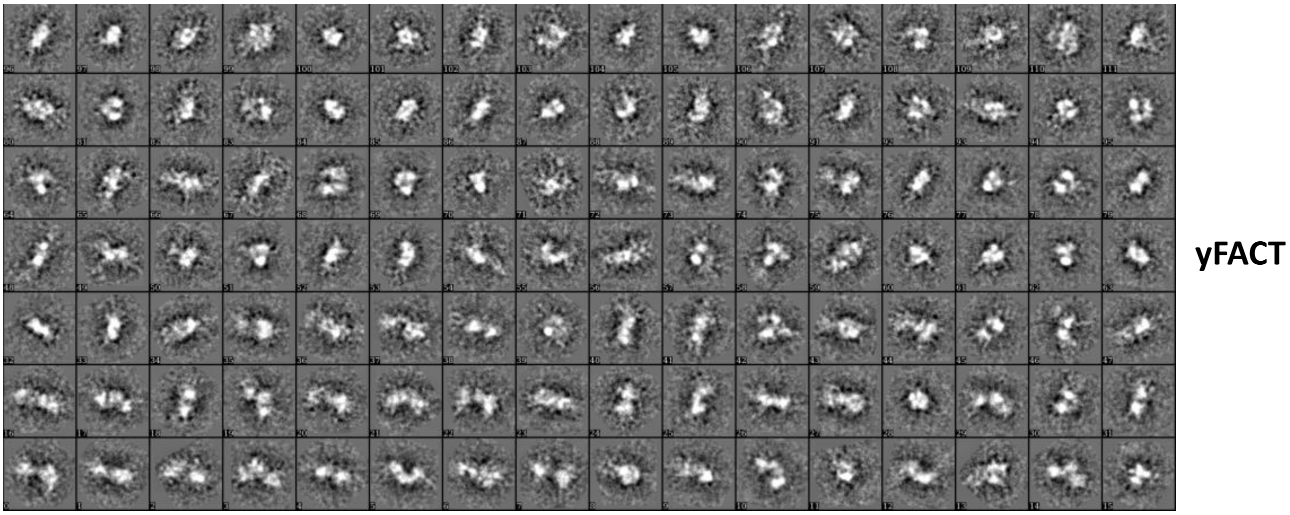

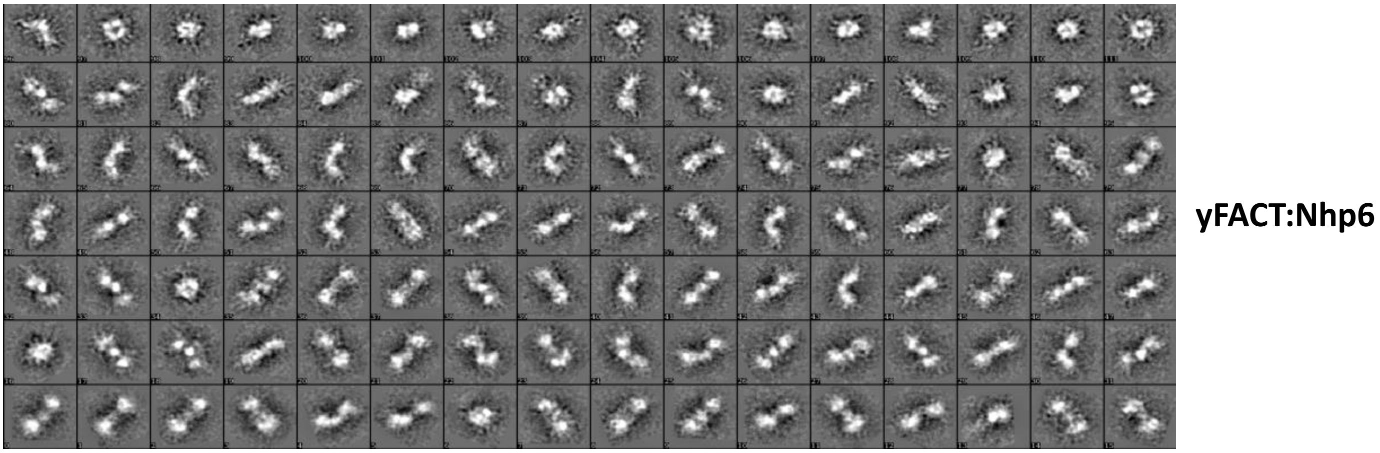

Supplementary Figure 4. 2D class-averages of Spt16/Pob3 (top) and Spt16/Pob3:Nhp6 complexes (bottom).

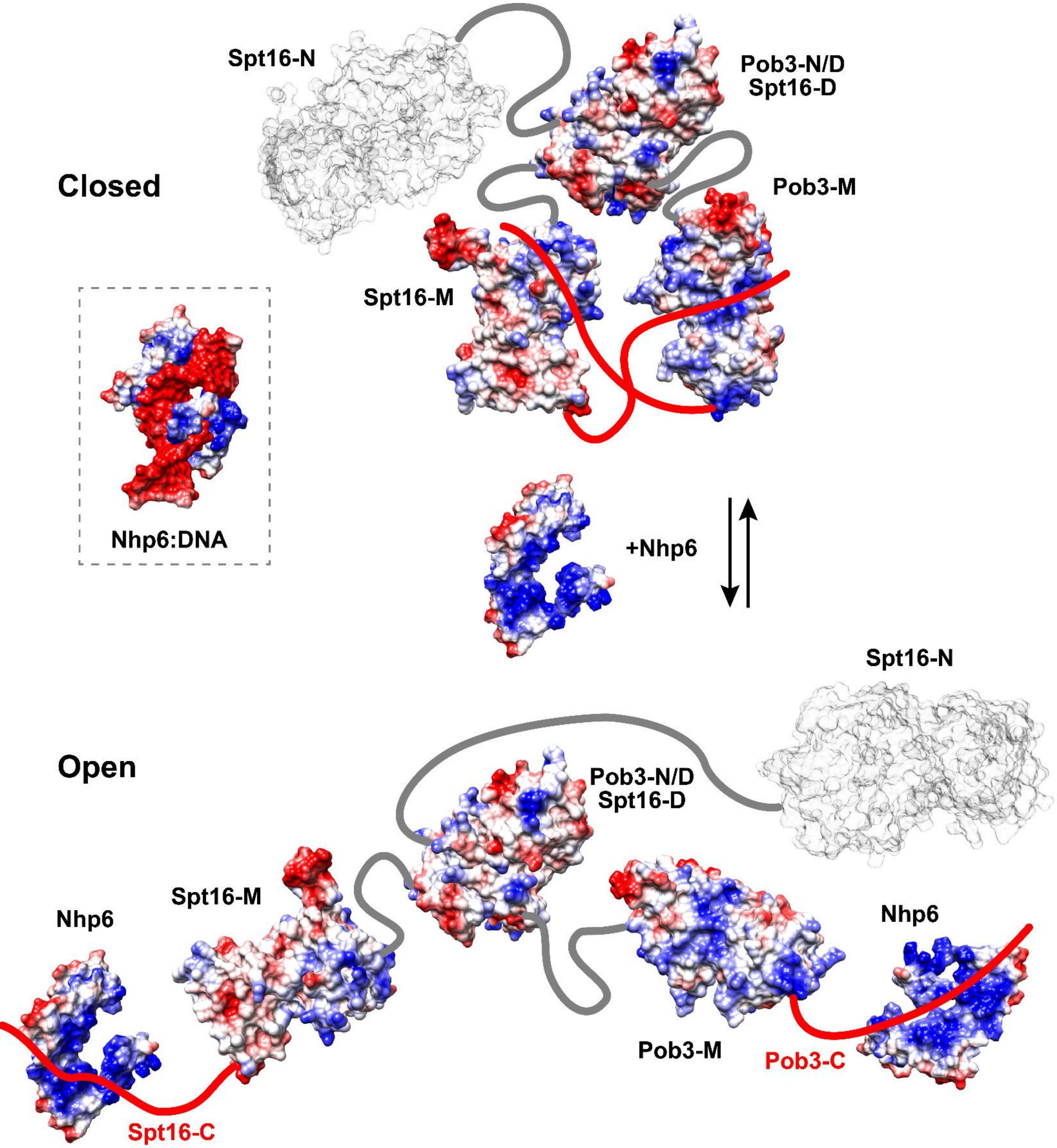

Supplementary Figure 5. A model of FACT and Nhp6:FACT complexes in closed and open states. Electrostatic potentials of the surfaces of relevant FACT domains and Nhp6:DNA generated in Chimera[25] using published PDB files for Spt16-N (3BIQ), Spt16-D:Pob3-N/D (4KHB), Spt16-M (4IOY), Pob3-M (2GCL), and Nhp6:DNA (1J5N) are shown (red = -10, blue = +10 kcal/mole at 298° K). We propose that the negatively charged Spt16-C and Pob3-C domains can interact with positively charged surfaces of either FACT domains or Nhp6, swapping binding partners between the closed and open FACT states with Nhp6 binding favoring the open state.

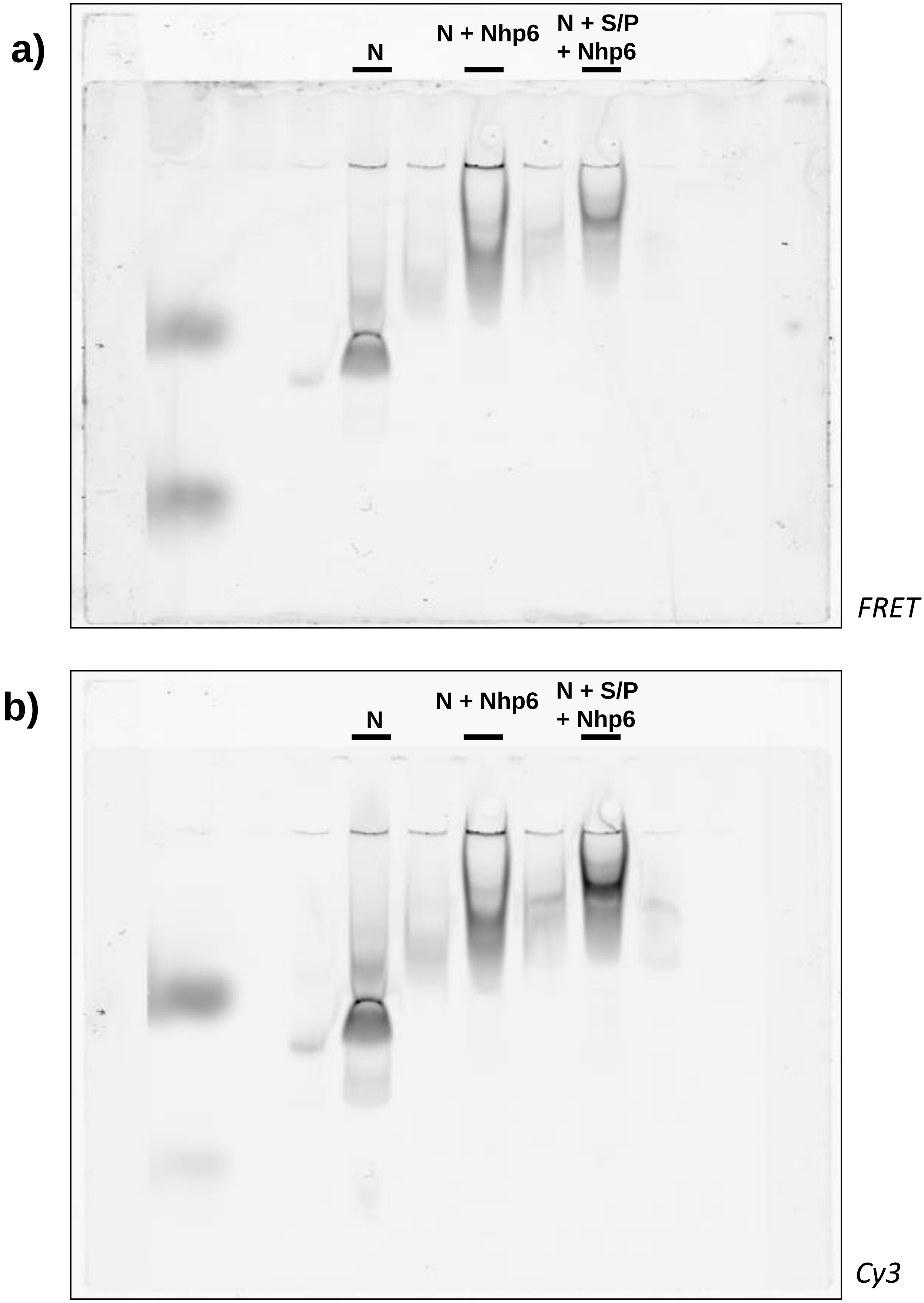

Supplementary Figure 6. Analysis of FACT-nucleosome complex by FRET-in-gel. Characterization of FACT:Nhp6 complexes containing fluorescently labeled N35/112 nucleosomes (N) by FRET (a) and Cy3 fluorescence (b). Complexes were separated by native PAGE, and the gel was analyzed as described in Methods.

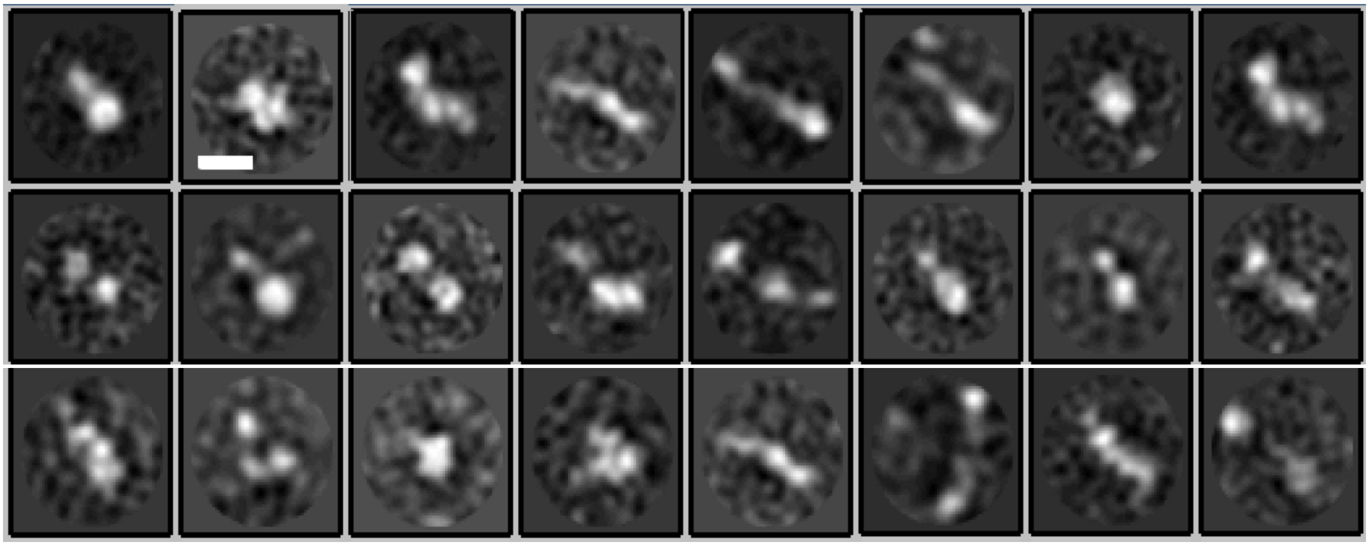

Supplementary Figure 7. 2D Class-averages of Spt16/Pob3:Nhp6:nucleosome complexes. Scale bar – 10 nm.

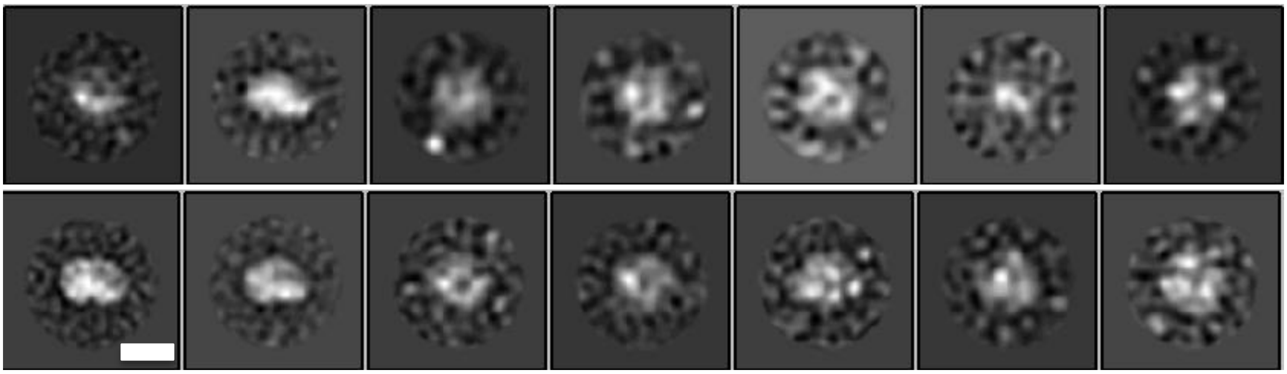

Supplementary Figure 8. 2D Class-averages of samples containing Nhp6 and nucleosome. Scale bar – 10 nm.

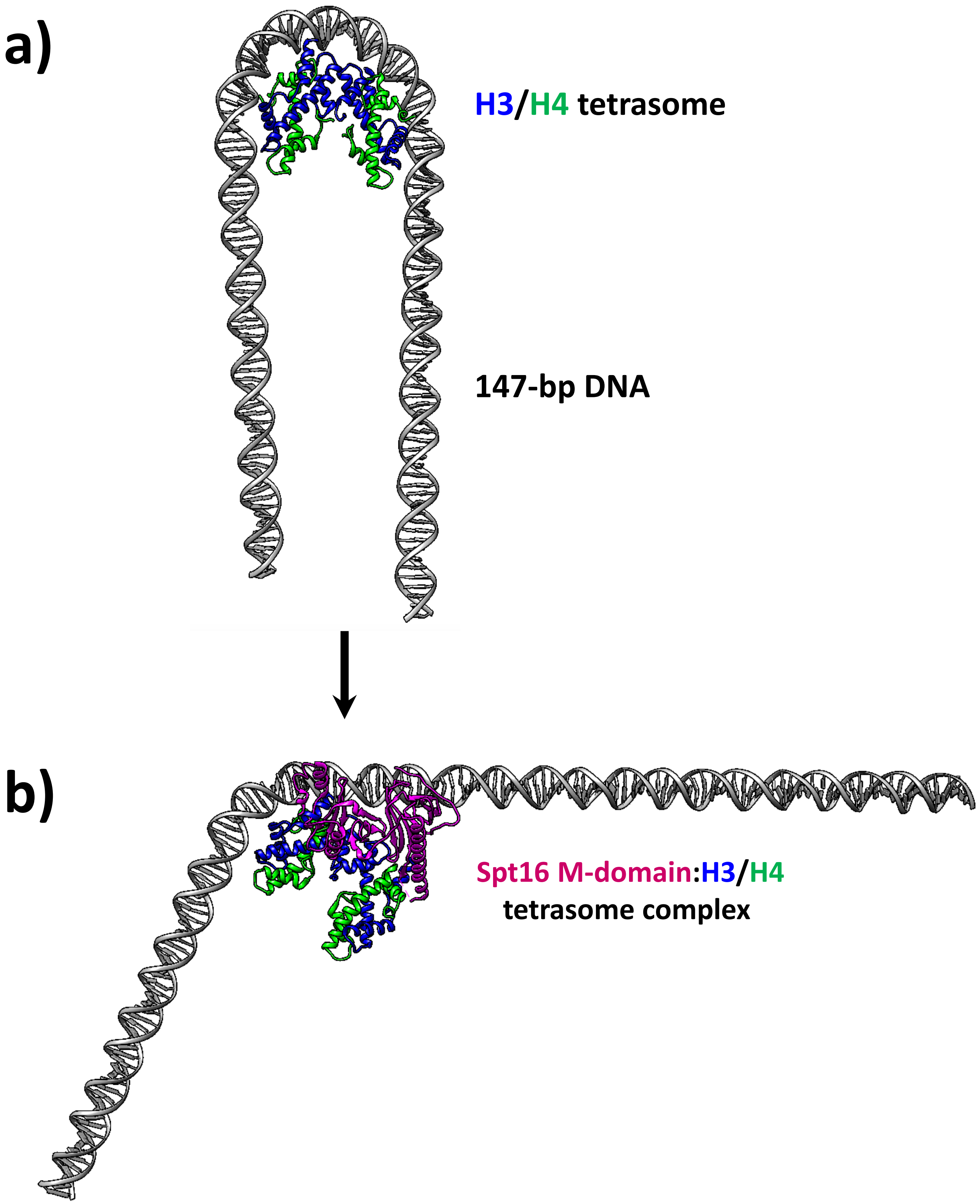

Supplementary Figure 9. Hypothetical model of a complex of tetrasome with M-domain of Spt16 subunit of yFACT. a) H3/H4 tetrasome structure. **b)** Binding of Spt16-M domain to H3/H4 tetramer is asymmetric and incompatible with DNA binding on the surface of the tetramer. To allow formation of the Spt16-M:H3/H4 tetramer complex, several DNA turns must be asymmetrically uncoiled from the surface of the tetramer.

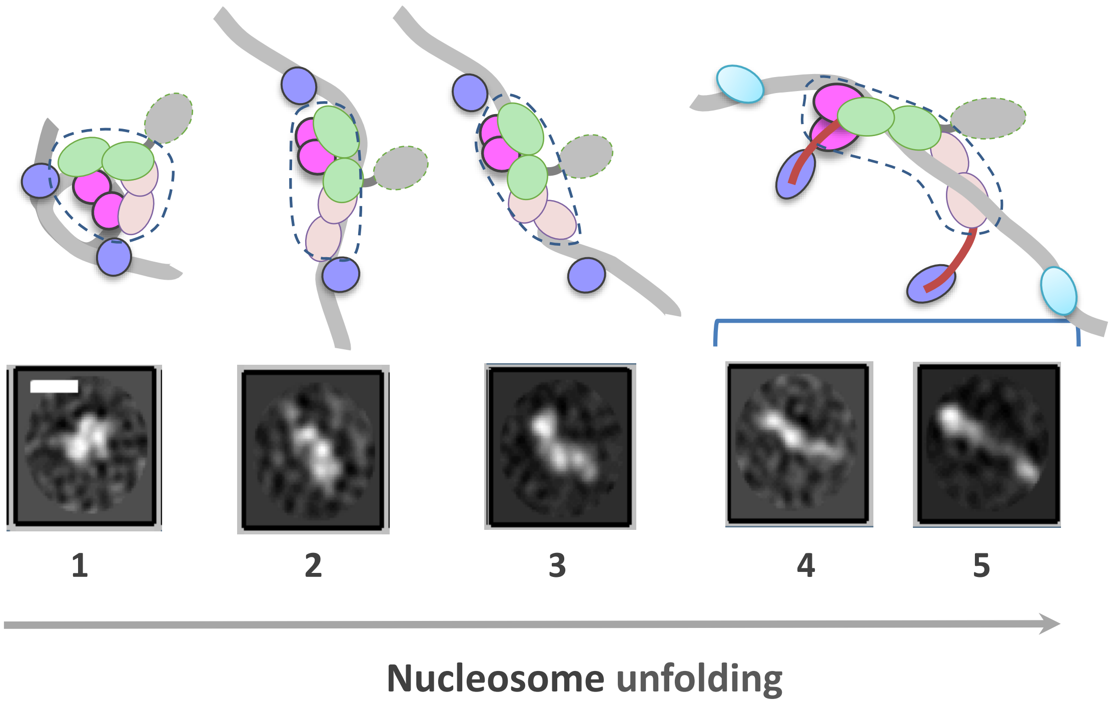

Supplementary Figure 10. The proposed sequential pathway of nucleosome unfolding by FACT:Nhp6. The structures of the intermediates from **Fig. 3e** were interpreted based on the assignment of the electron densities proposed in **Fig. 5b**. The resolved parts of the structures are shown by dashed lines. Other designations as in **Fig. 4b**.
