# Supplementary material for: Electron microscopy analysis of ATP-independent nucleosome unfolding by FACT
Source: Commun Biol. 2022 Jan 10;5:2. doi: 10.1038/s42003-021-02948-8 (PMC8748794; doi:10.1038/s42003-021-02948-8)
Supplement: Supplementary file 2 — Description of Additional Supplementary Files [file 42003_2021_2948_MOESM2_ESM.pdf]

Description of Additional Supplementary Files

File name: Supplementary Movie 1
Description: Flexibility of yFACT complex based on 2D classes of EM samples.

File name: Supplementary Movie 2
Description: Flexibility of yFACT:Nhp6 complex based on 2D classes of EM samples.

File name: Supplementary Data 1
Description: Quantitation of particles identified as closed (≤90°) or open (>90°) in samples with and without Nhp6.

File name: Supplementary Data 2
Description: Raw data for frequency distributions for nucleosomes N35/112 by the proximity ratios (EPR) in the absence or in the presence of Nhp6 and FACT.

File name: Supplementary Data 3
Description: Fractions of closed (90º) complexes in FACT:Nhp6:nucleosome and Nhp6:nucleosome complexes.
