# Supplementary figures and images for: Electron microscopy analysis of ATP-independent nucleosome unfolding by FACT
Source: Commun Biol. 2022 Jan 10;5:2. doi: 10.1038/s42003-021-02948-8 (PMC8748794; doi:10.1038/s42003-021-02948-8)

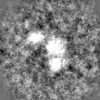

Supplement: Supplementary file 6 — Supplementary Movie 1 [file 42003_2021_2948_MOESM6_ESM.gif]

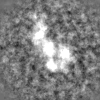

Supplement: Supplementary file 7 — Supplementary Movie 2 [file 42003_2021_2948_MOESM7_ESM.gif]
